# Effective degradation of DELTA pesticide in soil by an atmospheric-pressure cold plasma-DBD system

**DOI:** 10.1039/d5ra08882h

**Published:** 2026-01-12

**Authors:** Sushma Jangra, Abhijit Mishra, Ritesh Mishra, Shikha Pandey, Ram Prakash

**Affiliations:** a Department of Physics, Indian Institute of Technology Jodhpur Rajasthan 342030 India; b Interdisciplinary Research Division – Smart Healthcare, Indian Institute of Technology Jodhpur Rajasthan 342030 India ramprakash@iitj.ac.in

## Abstract

In protecting crops and increasing yields, the usage of pesticides, such as deltamethrin (DELTA), has increased due to rising worldwide food demand. However, the toxicity of DELTA, its limited biodegradability, and persistence are causing harm to the environment and also human health. There exist conventional soil remediation methods, but they are either costly, slow, or may cause secondary pollution, prompting interest in greener solutions. Atmospheric-pressure Cold Plasma (ACP) produced by dielectric barrier discharge (DBD) can offer an eco-friendly and innovative method for remediating pesticide-contaminated soil. In this study, an ACP-DBD plasma source was designed and developed and employed for the degradation of DELTA in soil. Cocopeat was used as a model soil to simulate field conditions. The effects of key operational parameters such as frequency, discharge voltage, treatment time, various pesticide concentrations, cocopeat soil pH and moisture were systematically evaluated to determine optimal conditions for maximum degradation efficiency. The onsite generation of reactive oxygen and nitrogen species within soil pores facilitated the effective degradation of DELTA, achieving removal efficiencies of up to 84.8% under optimized operational parameters. The detailed FTIR and GC-MS analysis further identified distinct degradation intermediates, supporting a mechanistic pathway predominantly driven by hydroxyl radicals and singlet oxygen (^1^O_2_). These findings are consistent with established plasma chemistry and underscore the oxidative transformation routes underlying pesticide breakdown. The results also highlight the potential of ACP-DBD as a green and effective technology for remediating pesticide-contaminated soils.

## Abstract 2

Degradation of deltamethrin in soil was achieved using an ACP-DBD system, where plasma-generated RONS interact with pesticide molecules, leading to molecular breakdown into less toxic intermediates and improved soil quality.

## Graphical abstract

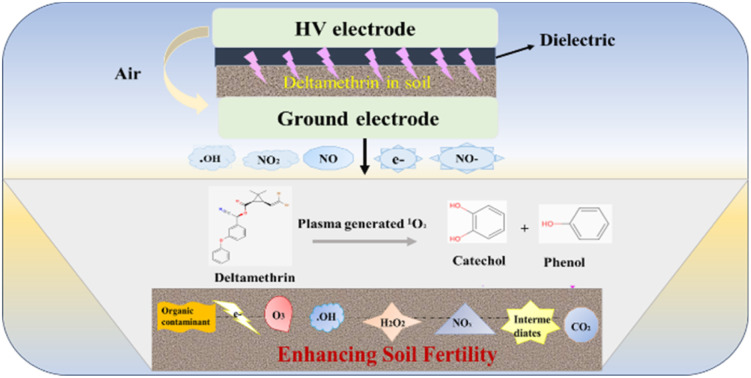

## 1. Introduction

Pyrethroid-based pesticides, especially Deltamethrin (DELTA), have become widely used due to their high efficacy, chemical stability, and affordability, making them a common choice for protecting crops from pests, insects, fungi, and weeds.^1,2^ However, the environmental persistence of DELTA poses a considerable challenge.^3^ Its half-life in cocopeat soil has been observed to range from 60 days to more than a year, indicating that its natural degradation occurs very slowly.^4^ Due to their high toxicity, low biodegradability, and carcinogenic and bioaccumulative qualities, the frequent and excessive use of such synthetic compounds adds to long-term ecological harm and puts human health at risk.^5^ Numerous soil remediation techniques, such as physical approaches, electron-Fenton processes, microwave remediation, microbial and phytoremediation, and conventional chemical treatments, have been developed to address these problems.^6–8^ Although these methods show potential, they are often constrained by lengthy treatment durations, high operational costs, and the risk of secondary pollution. Consequently, there is a growing interest in more efficient, eco-friendly, and rapid remediation technologies.

In recent years, Advanced Oxidation Processes (AOPs) have become a viable method for breaking down organic pollutants.^9^ Among these, non-thermal plasma-based AOPs offer unique advantages.^10^ Non-thermal atmospheric pressure plasma relies on high-energy electron–molecule interactions to produce a variety of reactive oxygen and nitrogen species (RONS), such as ozone (O_3_), atomic oxygen (O), hydrogen peroxide (H_2_O_2_), hydroxyl radicals (˙OH), and various charged species (*e.g.*, O_2_^−^, O_2_^+^, H_3_O^+^), in contrast to traditional methods that require chemical additives.^11,12^ These species provide a highly oxidative environment that can degrade stable and complex pollutants. Although non-thermal plasma has been extensively studied for air pollution and wastewater treatment, its use in soil remediation, especially for pesticide residues, remains relatively unexplored. Several recent studies have highlighted its potential, such as Zhan *et al.* (2018)^13^ achieved significant degradation of 200 mg kg^−1^ fluorene in cocopeat soil using a needle-plate pulsed corona discharge system within 60 minutes. Angelopoulos *et al.* (2018)^14^ demonstrated effective degradation of 10 mg kg^−1^ of atrazine using DBD plasma. Singh *et al.* (2017)^15^ reported the complete removal of 10 mg L^−1^ 2,4-dichlorophenoxyacetic acid (2,4-D) from aqueous media within 10 minutes using a multipin plane pulsed corona discharge configuration. Similarly, Lu *et al.* (2017)^16^ achieved a 70% degradation of phenanthrene after 40 minutes of plasma, and Wang *et al.* (2011)^17^ used a needle-plate technique to treat *p*-nitrophenol-contaminated cocopeat soil, resulting in a significant decrease in total organic carbon (TOC) and chemical oxygen demand (COD). Among plasma-based methods, Atmospheric Cold Plasma produced *via* Dielectric Barrier Discharge (ACP-DBD) stands out for its simplicity, operation at atmospheric pressure, low-temperature processing, and absence of chemical additives.^16,18^ Despite these advantages, the specific pathways, interaction between RONS and soil-bound contaminants, and the influence of soil matrix properties remain poorly understood.

In this study, we established a systematic cold atmospheric plasma approach for soil remediation by developing and implementing an ACP-DBD system specially designed for *in situ* treatment. Cocopeat was selected as the model soil matrix to closely mimic uncontaminated soil conditions while avoiding interference from pre-existing chemical residues. To evaluate the degradation potential, the cocopeat was deliberately spiked with DELTA pesticide. Then the spiked pesticides were exposed to the developed ACP-DBD system to examine the efficiency of the system to remove DELTA model from cocopeat soil. The study has also systematically evaluated how major operational factors -such as discharge voltage and frequency, varying pesticide dosages, cocopeat soil pH and moisture, and treatment time affect degradation performance and energy use, with the goal of determining the most efficient operating conditions. To characterize the plasma, Optical Emission Spectroscopy (OES) was employed to monitor the generation of reactive plasma species during treatment. Furthermore, Gas Chromatography-Mass Spectrometry (GC-MS) and Fourier-Transform Infrared Spectroscopy (FTIR) analysis were done to measure DELTA degraded metabolites, subsequently these intermediates compound was used to derive the possible mechanism of DELTA degradation under plasma-generated RONS. To validate the toxicity of intermediate compounds and its comparison with the parent molecule, a toxicity estimation tool was incorporated. To the best of our knowledge, this is the first study to simulate the pesticide degradation in soil using plasma-generated RONS and confirm the effectiveness of the ACP-DBD plasma device to reduce the pesticide content in soil. Overall, this research highlights the potential of ACP-DBD to serve as a chemical-free and sustainable alternative for *in situ* soil remediation, offering new insights into plasma–soil interactions and paving the way for improving soil health, reducing contaminants, and promoting cleaner agriculture practices.

## 2. Materials and methods

### 2.1 Experimental set-up

**Fig. 1:**
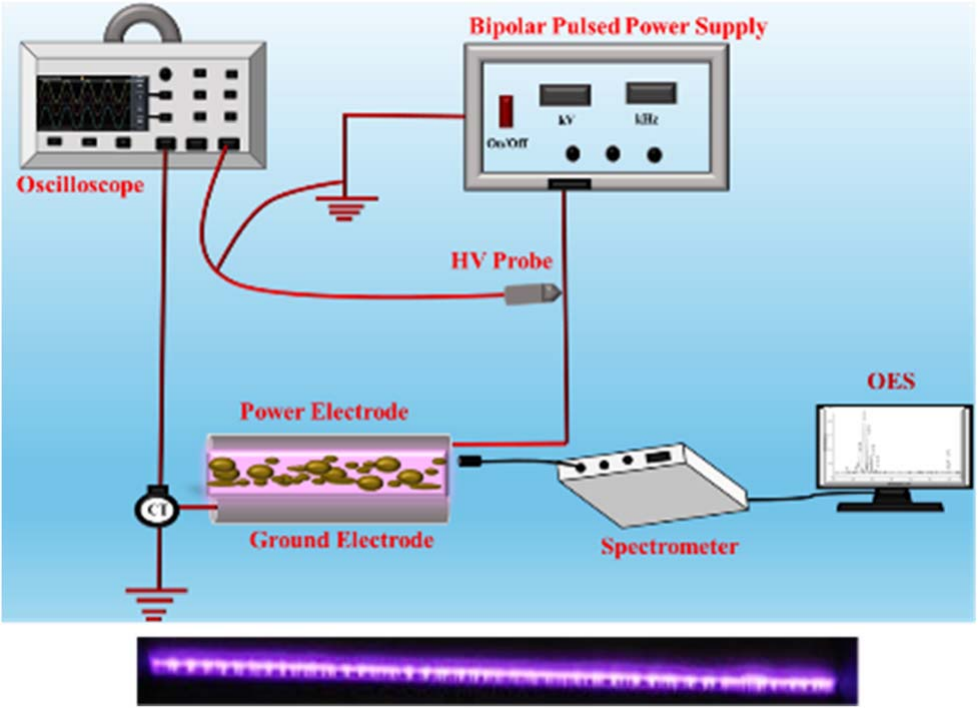
Schematic diagram of the experimental setup (ACP-DBD) device for the treatment of DELTA-contaminated soils. The image at the bottom shows the active plasma zone formed between the electrodes.

DELTA degradation in cocopeat soil was investigated using an experimental set-up shown in Fig. 1, which is based on an ACP-DBD system, comprising a cylindrical electrode configuration with both stationary and rotating components. The active plasma zone responsible for cocopeat soil treatment is shown in the figure. A detailed description of this device was given in previous papers.^19–21^ The system consisted of a variable bipolar pulsed power supply with the following specifications: 1–10 kV peak voltage, 5–40 kHz frequency range, 2 µs pulse width, and a maximum peak current of 1 A. This power supply provided precise control over the energy input and the reactive species generated during discharge. Discharge voltage and current were measured using a high-voltage probe (Tektronix P6015A) and a current transformer (Pearson™ model 110), respectively. Signal acquisition and waveform monitoring were performed *via* a digital oscilloscope (Tektronix MDO3014, 100 MHz bandwidth). The energy consumed is calculated by using the following formula^22^1
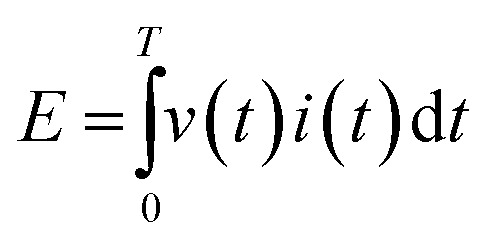
where *E* is the energy consumed, *T* is the period of oscillation, *v(t)* is the instantaneous voltage, and *i(t)* is the current. The discharge power consumed in the ACP-DBD is estimated using the following relationship.^23^2
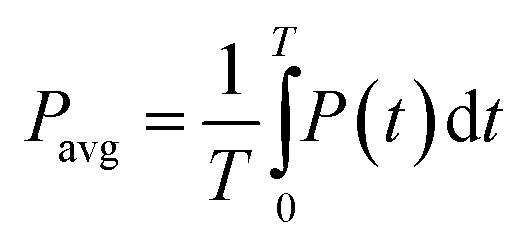


The electron density was estimated from the voltage–current (*V*–*I*) characteristics using the methodology described elsewhere.^21^ To characterize the plasma and identify the active species involved in pesticide degradation, OES was carried out using a spectrometer (Andor Shamrock SR-500i-B1) with a spectral resolution of 0.1 nm across the 200–900 nm wavelength range. The emission was collected *via* a 200 µm optical fiber (Ocean Optics), positioned 3 cm from the plasma discharge region. This configuration allowed for a thorough evaluation of plasma-induced degradation mechanisms under air circumstances by ensuring effective and regulated plasma interaction with pesticide-contaminated cocopeat soil.

### 2.2 Materials and methods

#### 2.2.1 Sample preparation

Deltamethrin (DELTA) was purchased from Tokyo Chemical Industry Co., Ltd, and its physicochemical properties are mentioned in Table 1,^24^ while all organic and inorganic reagents used were of analytical grade and procured from Sigma-Aldrich. Cocopeat, purchased locally from Jodhpur, served as the soil substrate. Prior to contamination, the cocopeat was air-dried for two days, sieved through a 20-mesh standard sieve, then washed for 12 hours using an *n*-hexane and acetone (v/v = 1 : 1) mixture to remove residual impurities that could interfere with subsequent analyses. Before use, the cleaned cocopeat was then air-dried. To prepare DELTA-contaminated cocopeat samples. 10 grams of cocopeat were thoroughly mixed with 10 microliters of DELTA dissolved in 10 milliliters of isopropanol. The mixture was then placed in a fume hood to ensure complete evaporation of the isopropanol, resulting in a homogeneous pesticide concentration of approximately 1 mg per kg of cocopeat. To create samples with varying concentrations, the amount of DELTA added was adjusted accordingly, following the same preparation method. The initial concentrations tested included 10, 20, and 30 microliters of DELTA per 10 grams of cocopeat. Before analytical procedures, any impurities were removed from the samples to prevent interference during high-performance liquid chromatography (Waters, Model 2489) and gas chromatography-mass spectrometry (GC-MS) (Shimadzu QP-2010 Plus with Thermal Desorption System TD 20) analyses. The concentration of DELTA in the cocopeat was determined using ultraviolet-visible (UV-Vis) spectrophotometry (PerkinElmer, USA) at a wavelength of 286 nm. FTIR spectra were acquired for plasma-treated cocopeat utilising attenuated total reflectance *via* FTIR spectroscopy.^25^ FTIR (Vertex, 70 V + PMA50) with a range of 400–4000 cm^−1^ was utilised to distinguish DELTA peaks.

**Table 1:** Physicochemical properties of deltamethrin in soil

Pesticide name	Deltamethrin
Physical appearance	Off-white solid powder (technical grade)
Molecular formula	C_22_H_19_Br_2_NO_3_
Molecular weight (g mol^−1^)	505.2
Wavelength (nm)	286
Density (g cm^−3^)	0.550
Structure of deltamethrin (DELTA)	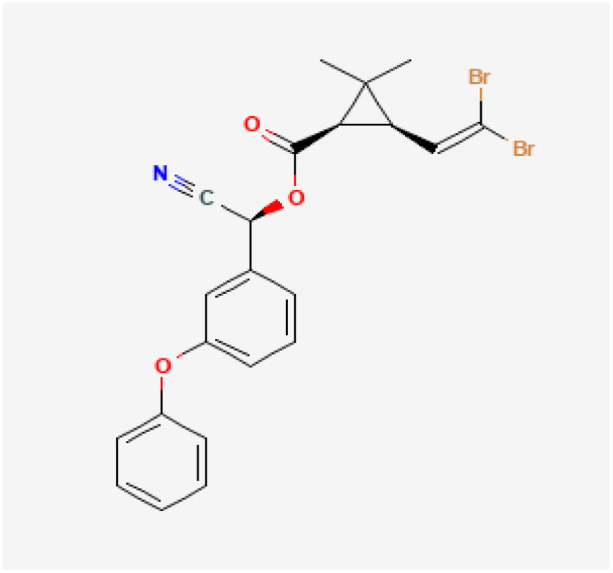
pH	5.5
Electrical conductivity	1 mS cm^−1^
Moisture content	10.86

#### 2.2.2 Extraction and analysis

DELTA was extracted from cocopeat soil samples after each ACP-DBD treatment using 20 mL of acetonitrile, which was added to 5 g of cocopeat soil. The mixture was shaken at 220 rpm at 20 °C for 60 minutes, followed by sonication at 25 °C for 30 minutes. The sample was then centrifuged at 6000 rpm for 20 minutes. The supernatant was filtered through 0.45 µm membranes, and a 25 µL aliquot was analyzed *via* HPLC (Waters Model 2489) with a mobile phase of acetonitrile: water (20 : 80 v/v, 1.0 mL min^−1^ flow rate) and UV-Vis detection at 286 nm. A linear calibration curve (*Y* = 51 758*x* − 27 908) validated quantification. Cocopeat soil moisture content was determined using a standard weighing method. The cocopeat soil samples were dried at 105 ± 2 °C for approximately 2 hours until a constant weight was achieved. The difference in the cocopeat soil's weight before and after drying was used to calculate the moisture content using the following formula:3
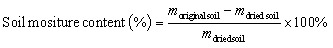


By-products were characterized using FTIR (Bruker Vertex 70 v) and GC-MS (Shimadzu QP-2010 Plus with Thermal Desorption System TD 20), while degradation efficiency (%) was calculated as^14^4
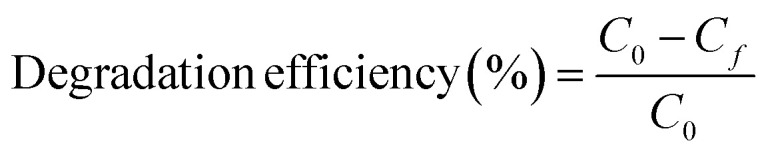
where *C*_0_ and *C*_*f*_ are the initial and final concentrations of the DELTA pesticide.

Energy efficiency (*E*) was derived as^26^5
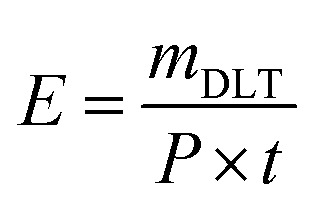
where *m*_DLT_ is the mass of DELTA degraded at treatment time *t*, *P* as discharge power, and *t* as treatment time. The dielectric barrier discharge system achieved rapid dissociation, with optimal degradation observed at 15 minutes of plasma exposure.

### 2.3 Biotoxicity evaluation

The toxicity of DELTA and its degradation intermediate products was evaluated by using the US Environmental Protection Agency-recommended software (a) Toxicity Estimation Software Tool (TEST).^14,27^

## 3. Results and discussion

### 3.1 Electrical characteristics of the ACP-DBD device

**Fig. 2:**
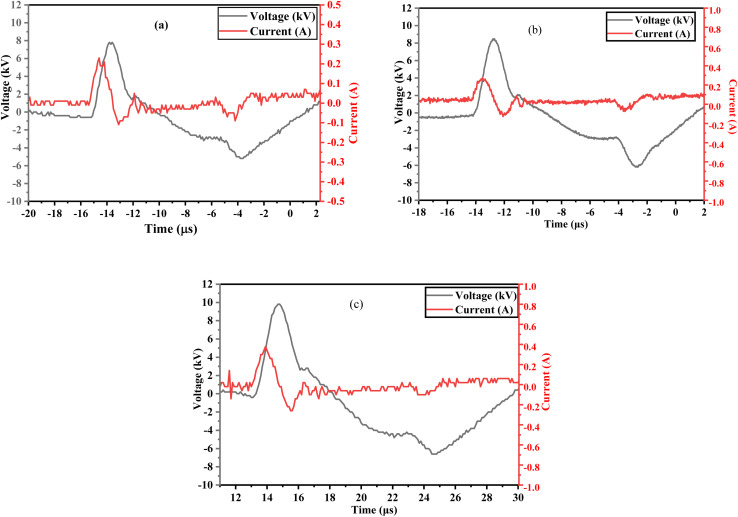
*V*–*I* characteristics of ACP-DBD at applied voltages (a) 8 kV/20 kHz, (b) 9 kV/20 kHz and (c)10 kV/20 kHz.

To evaluate the electrical characteristics of the ACP-DBD reactor used in this study, the instantaneous voltage and current waveforms were recorded during cocopeat soil treatment at different applied voltages of 8 kV, 9 kV and 10 kV, all operated at a fixed frequency of 20 kHz. The corresponding waveforms are illustrated in Fig. 2a–c. The discharge power measured at 8 kV, 9 kV, and 10 kV was approximately 4.3 W, 5.4 W, and 9.9 W, respectively. As the voltage increased, both the peak current and discharge power showed a corresponding increase.

### 3.2 Plasma reactive species identification and quantification

**Fig. 3:**
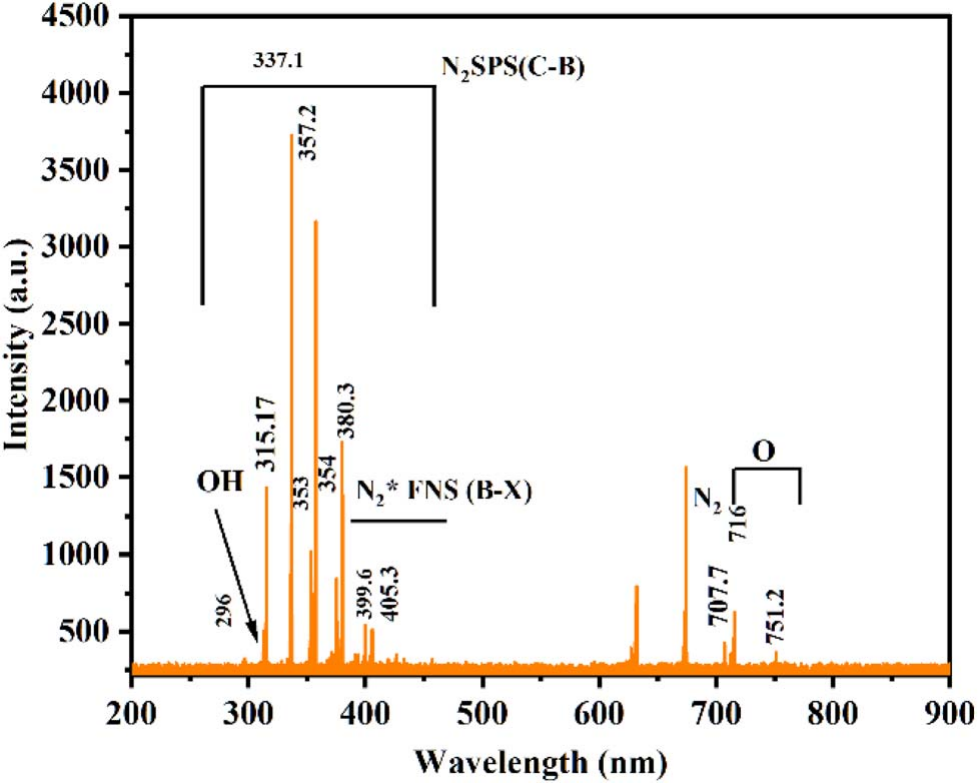
Optical emission spectra of air plasma obtained from the DBD reactor.

ACP treatment under atmospheric air generates a variety of RONS through the dissociation of nitrogen, oxygen, and water molecules in the discharge zone, which can play a critical role in degrading organic contaminants like DELTA in cocopeat soil.^28^ The ACP-DBD plasma device was utilized accordingly for producing micro-discharges that facilitated *in situ* remediation. The excited species produced in plasma were investigated by the OES technique, and a typical spectrum in the 200–900 nm wavelength range is shown in Fig. 3. N_2_ second positive system (SPS) (C^3^П_u_–B^3^П_g_) peaks at (296, 315.7, 337, 353, 357.2, and 380.3) and N_2_ first positive system (FPS) (B^3^П_g_–A^3^П_u_^+^) peaks in the range (500–700) nm. Also, 
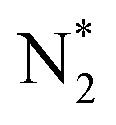
 first negative system (FNS) emissions at wavelengths of (399.6 and 405) nm. O(i) also get confirmed at wavelengths 716 and 752 nm. The generation of such reactive oxygen and nitrogen species through non-thermal plasma discharges has a beneficial impact on seed germination and plant growth. A detailed explanation of these effects is provided subsequently.

### 3.3 Effect of voltage, frequency, and various concentrations on DELTA degradation

**Fig. 4:**
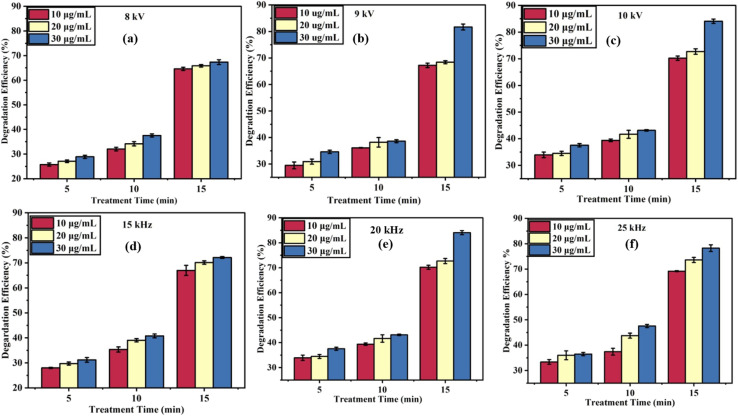
Degradation efficiency of DELTA at different voltages (a–c) and frequencies (d–f) for various concentrations and treatment durations.

To optimize the ACP-DBD degradation process of DELTA, various reaction parameters were systematically investigated. The high voltage generated between the electrodes led to the formation of active radicals, which were responsible for the decomposition of organic pollutants.^29^ The degradation efficiency of DELTA was evaluated under varying operational parameters, including discharge voltage (8, 9 and 10 kV), frequency (15, 20, and 25 kHz) and various pesticide concentrations (10, 20, and 30 µg mL^−1^) as shown in Fig. 4a–f. The findings showed a consistent increase in degradation efficiency with prolonged plasma exposure. At 8 kV, the degradation efficiencies after 15 minutes were approximately (64.46 ± 0.65)%, (66.17 ± 0.73)%, and (67.93 ± 0.67)% for 10, 20, and 30 µg mL^−1^ concentrations, respectively (Fig. 4a). Increasing the voltage to 9 kV further enhanced degradation, reaching (67.89 ± 0.52)%, (69.06 ± 0.83)%, and (81.70 ± 0.53)% for the same concentrations (Fig. 4b). The maximum removal efficiencies were recorded at 10 kV (Fig. 4c), with values of (70.50 ± 0.64)%, (73.13 ± 0.66)%, and (84.85 ± 0.98)%, highlighting that higher voltage intensifies the generation of reactive species, thereby promoting pesticide decomposition. A higher discharge voltage produces a stronger electric field and delivers greater power to the DBD system, leading to an increased space charge density, more intense UV emission, and enhanced production of reactive species. These combined effects significantly promote the degradation efficiency of DELTA.^30^

Another important factor in the degradation process is frequency. At 10 kV and 15 kHz, the efficiencies were moderate, reaching (68.12 ± 0.23)%, (70.94 ± 1.02)%, and (72.23 ± 2.02)% for 10, 20, and 30 µg mL^−1^, respectively (Fig. 4d). Increasing the frequency to 20 kHz (Fig. 4e) led to a significant improvement, achieving (70.50 ± 0.64)%, (73.13 ± 0.66)%, and (84.85 ± 0.98)%. However, a slight decline was observed at 25 kHz (Fig. 4f), with degradation efficiencies of (69.12 ± 0.94)%, (73.82 ± 0.77)%, and (78.59 ± 0.38)%, suggesting a potential saturation or inhibitory effect at higher frequencies. This minor decrease could be attributed to the accumulation of oxidation by-products on the cocopeat soil surface, which may hinder the interaction between DELTA in deeper layers and reactive plasma species. A comparable behaviour was also reported for *p*-nitrophenol-contaminated cocopeat soil treated with pulsed corona discharge plasma.^31^

**Fig. 5:**
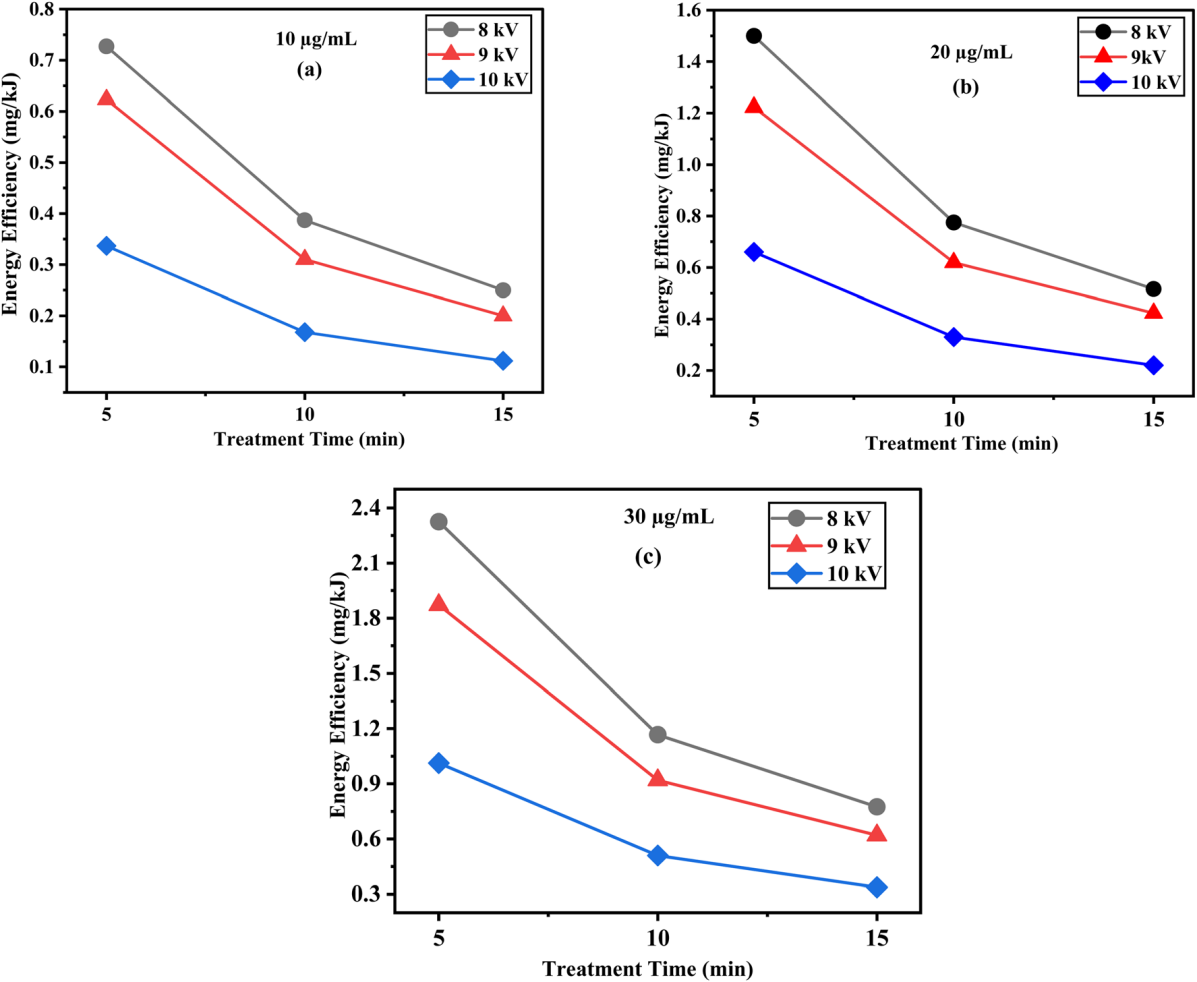
Energy efficiency of DELTA degradation as a function of plasma treatment time under different applied voltages at various concentrations (a) 10, (b) 20, (c) 30 µg mL^−1^.

The energy efficiency of DELTA degradation was further evaluated at discharge powers of 4.3 W (8 kV), 5.38 W (9 kV), and 9.88 W (10 kV) with various DELTA concentrations and treatment times as shown in Fig. 5. At a DELTA concentration of 30 µg mL^−1^ with 5 minutes of treatment, the corresponding energy efficiencies were 2.3 mg kJ^−1^, 1.8 mg kJ^−1^, and 1.03 mg kJ^−1^, respectively. Obviously, at each time point, lower voltages (8 kV) exhibited higher energy efficiency compared to 9 and 10 kV. At 5 minutes of treatment time, the energy efficiency for 30 µg mL^−1^ was highest at 8 kV (2.3 mg kJ^−1^), followed by 9 kV (1.9 mg kJ^−1^) and 10 kV (1.0 mg kJ^−1^). Similar trends were observed for 20 µg mL^−1^ and 10 µg mL^−1^ concentrations, where the highest efficiency was again achieved at 8 kV. According to this pattern, higher voltages increase degradation and produce more reactive species, but they also consume more energy, thus lowering energy efficiency. Overall, the findings highlight a trade-off between degradation performance and energy cost, with lower voltages and shorter treatment times offering optimal energy efficiency for plasma-based DELTA remediation.

### 3.4 Effect of pH and cocopeat soil moisture content on DELTA degradation efficiency

**Fig. 6:**
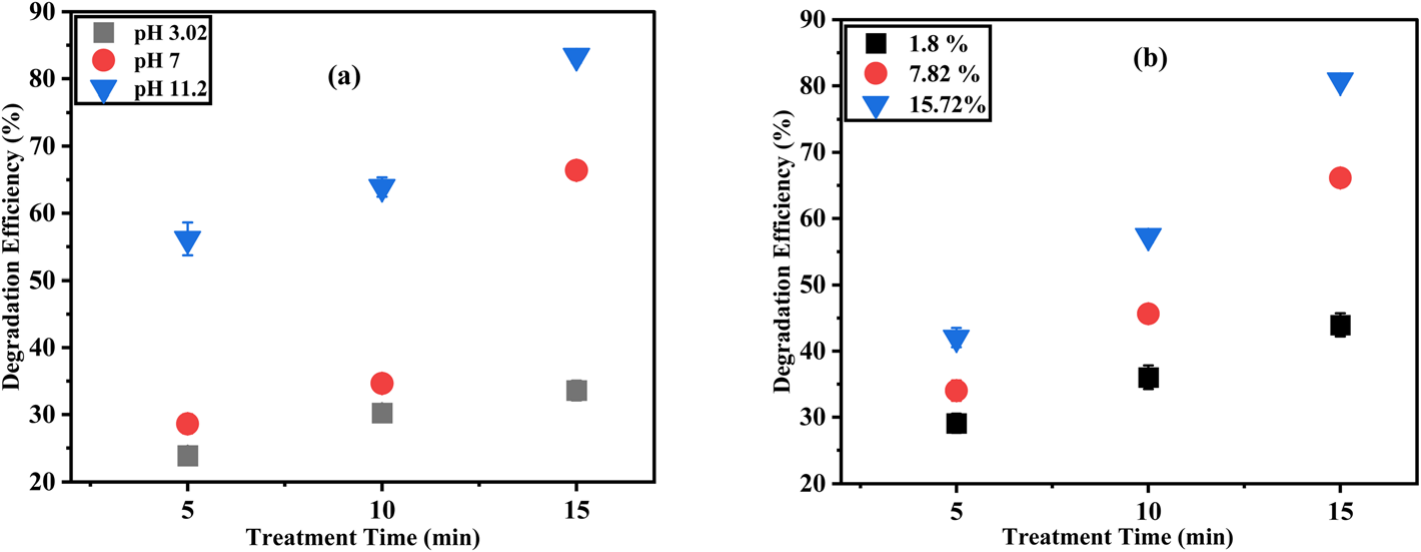
Effect of (a) cocopeat soil type; (b) cocopeat soil moisture at different plasma treatment time (cocopeat soil mass: 5 g, DELTA concentration: 30 microliter concentration, 20 kHz, 10 kV).

Soil characteristics such as pH, water content, organic matter and initial concentration of contaminants significantly influence the effectiveness of pollutant degradation during plasma-based treatment.^32^ Among these factors, soil pH affects the dissociation and adsorption behaviour of organic compounds.^33^ In this study, DELTA contaminated cocopeat soil was treated using the ACP-DBD system under acidic (pH 3.02), neutral (pH 7) and alkaline (pH 11.2) conditions for treatment durations of 5, 10, and 15 minutes. The detailed preparation method for the pH-adjusted soil samples is provided in SE.1. As illustrated in Fig. 6a, alkaline conditions significantly enhanced DELTA degradation, with the highest degradation efficiency reaching approximately (84.11 ± 1.48)% after 15 minutes. However, neutral and acidic conditions led to reduced efficiencies of (64.59 ± 0.66)% and (57.01 ± 0.95)%, respectively, at the same duration. The production of ROS, such as ˙OH and O_3_, which are essential for oxidative degradation of pesticide molecules, is responsible for enhanced performance in an alkaline environment. In acidic conditions, specific surface functional groups present in cocopeat can limit the formation of these reactive species, thereby lowering the overall degradation rate. On the other hand, alkaline conditions promote ozone decomposition and increase ROS generation, facilitating efficient contaminant breakdown.

The presence of H_2_O in the air also contributes to the production of hydroxyl radicals through several plasma-induced reactions,^34^ including:

6 O_2_ + e^−^ → 2O˙ + e^−^

7 2O˙ + 2O_2_ → 2O_3_

8 O_3_ + H_2_O_2_ → ˙OH + HO_2_ + O_2_

9 e^−^ + O_3_ → e^−^ + O + O_2_

10 N + O_2_ → NO + O

11 N + O_3_ → NO + O_2_

12 O + NO_2_ → NO + O_2_

Further studies have shown that acidic conditions can hinder the formation of hydroxyl radicals due to the interaction of surface functional groups, thereby reducing the removal efficiency of contaminants such as DELTA.^35^ In alkaline cocopeat soils, ozone degrades more efficiently than in acidic conditions, which supports the formation of additional reactive species. Studies also indicate that hydroxyl radical generation increases significantly as the pH of cocopeat rises from 3.1 to 8.9.^36^ Since both hydroxyl radical production and ozone breakdown are important components of oxidative degradation, their enhancement under higher pH conditions (alkaline conditions) significantly boosts pollutant removal. As a result, this method shows great potential for removing organic contaminants in cocopeat soils over a wide pH range, performing noticeably better in alkaline conditions because of more advantageous secondary reaction pathways.^37^

Soil moisture plays a crucial role in determining pollutant mobility and the efficiency of various remediation approaches. To investigate this effect, ACP-DBD treatments were carried out on DELTA-contaminated cocopeat at three moisture levels- 1.8%, 7.82%, and 15.72% using the optimized operating conditions (applied voltage 10 kV, 20 kHz frequency and 30 µL DELTA). Fig. 6b shows the impact of moisture content on DELTA degradation efficiency in cocopeat soil. The highest moisture content (15.72%) resulted in the most effective degradation, reaching (82.09 ± 1.75)% after 15 minutes. The lowest moisture (1.8%) achieved only (43.23 ± 1.79)% while moderate moisture content (7.82%) resulted in (57.48 ± 1.43)% degradation. These results imply that the presence of water facilitates the formation of reactive species such as ˙OH and H_2_O_2_, which accelerate the degradation process. However, excessively high moisture content can lead to pore blockage in the cocopeat structure, which could reduce the diffusion of reactive plasma species.^38^ Increased moisture enhanced radical production and transport throughout the tested range, supporting the degradation process. The initial concentration of pollutants also has a significant impact on the degradation results. The efficiency of degradation decreases as the concentration of DELTA increases because there are more contaminant molecules than plasma-induced reactive species. This inverse relationship shows that the mechanism is more efficient at lower concentrations, which is significant given that real-world agricultural soils typically contain pesticides at trace levels.^38,39^

### 3.5 HPLC analysis for DELTA degradation

**Fig. 7:**
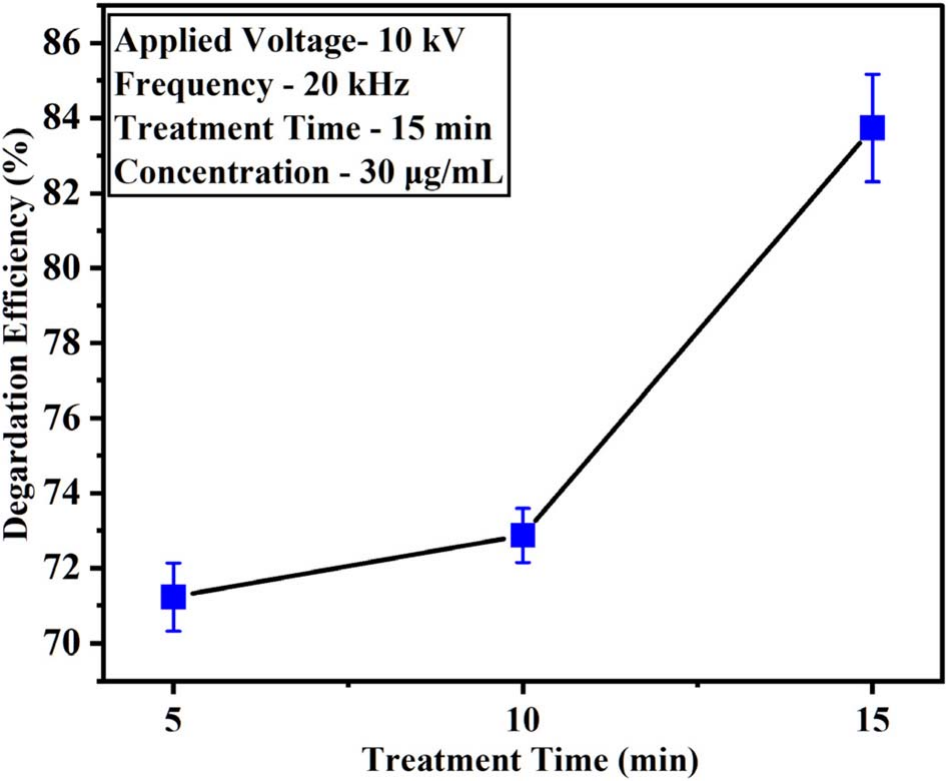
Degradation efficiency of DELTA pesticide.

The HPLC analysis was used to confirm the degradation of DELTA under ACP-DBD, as shown in Fig. SE.2. To evaluate the efficacy of plasma treatment, the maximum concentration of DELTA (30 µg mL^−1^) was used, and degradation efficiency was measured at different treatment durations (5, 10, and 15 minutes). As shown in Fig. 7, plasma exposure led to a significant reduction in pesticide concentration, with degradation efficiencies of (71.22 ± 0.90)% at 5 minutes, (72.87 ± 0.72)% at 10 minutes, and a significant increase to (83.98 ± 1.43)% at 15 minutes. This progressive enhancement in degradation is attributed to the intensified interaction of RONS, particularly hydroxyl radicals (˙OH), ozone (O_3_), and nitrogen oxides (NO_*x*_), generated by the ACP-DBD system. These results indicate that longer plasma treatment durations promote more efficient pesticide breakdown, reinforcing the potential of atmospheric cold plasma as a sustainable and environmentally friendly approach for pesticide remediation.

### 3.6 FTIR analysis for DELTA degradation

**Fig. 8:**
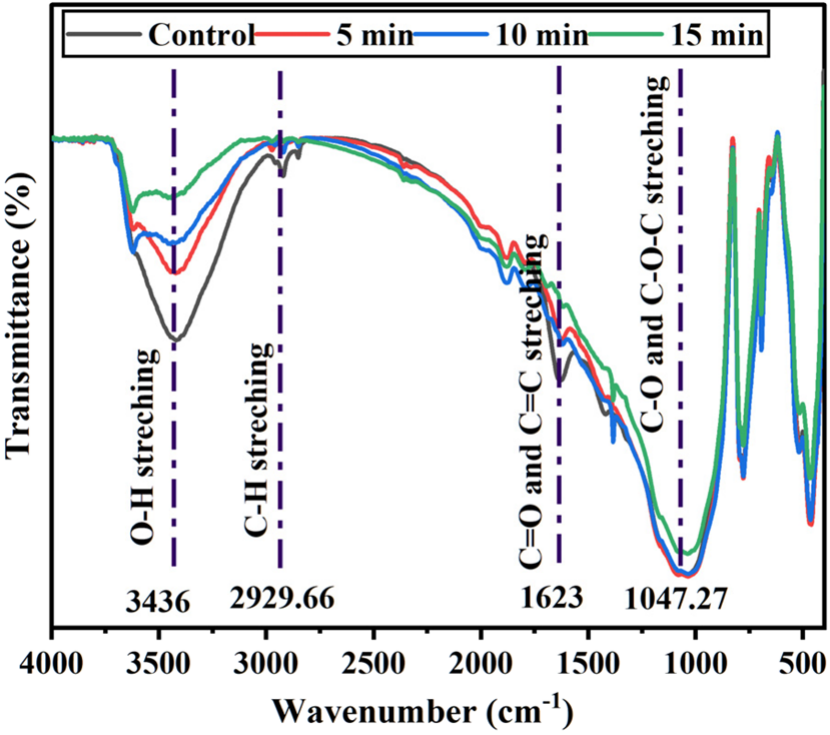
FTIR of untreated and treated cocopeat soil at various times (10 kV/20 kHz).

The FTIR spectra of untreated and ACP-DBD treated cocopeat soil samples are depicted in Fig. 8. The untreated sample spectra exhibited characteristic absorption bands of the functional groups associated with intact DELTA. A broad absorption band at 3436 cm^−1^, corresponding to O–H stretching, indicates the presence of hydroxyl groups, possibly from environmental moisture or minor hydroxyl substitution.^40^ A peak at 2929.66 cm^−1^ is indicated to C–H stretching vibrations, typically observed in aliphatic hydrocarbons. The absorption near 1623 cm^−1^ represents C=O stretching (carbonyl groups) and C=C stretching of the aromatic ring, respectively. The region around 1047.27 cm^−1^ corresponds to C–O and C–O–C stretching, indicating the presence of ester and ether linkages in the molecular structure.^41–43^ After ACP-DBD plasma treatment, significant spectral changes were observed across all exposure durations (5, 10, and 15 minutes), reflecting progressive structural degradation of DELTA. As treatment time increased, the intensity of the O–H and C–H stretching bands decreased, indicating oxidative degradation of hydroxyl and aliphatic chains, likely caused by plasma-generated ROS, such as O, ˙OH, O_3_, and NO_*x*_. The reduction in O–H band intensity (3436 cm^−1^) specifically reflects the oxidation of aliphatic chains and hydroxyl groups, consistent with literature reports where DBD plasma was shown to reduce hydrophobic moieties and increase the hydrophilicity of cocopeat soil organic matter.^44^

The decline in the C=O and C=C absorption band at 1623 cm^−1^ indicates disruption of aromatic and carbonyl structures, possibly due to oxidative ring-opening or fragmentation by reactive nitrogen species. The attenuation and slight shift in the C–O and C–O–C bands at 1047.27 cm^−1^ suggest cleavage of ester and ether linkages, likely *via* radical-induced reactions or hydrolysis. Supporting evidence from recovered organic fractions showed additional bands, including =CH stretching (3143.40–3097.12 cm^−1^), C–H stretching (3008.41–2996.84 cm^−1^), a peak at 2360.40 cm^−1^ corresponding to C≡N stretching, shifts in C–O stretching from 1068.37 to 1022.09 cm^−1^, and changes in C–Br from 659.54 to 632.54 cm^−1^, indicating complex degradation of halogenated and nitrogen-containing moieties.^45,46^ These spectral shifts collectively confirm that ACP-DBD treatment alters the structural composition of DELTA through oxidative bond cleavage and formation of more polar degradation products. The degree of these chemical modifications increased with plasma treatment duration, consistent with UV-Visible spectroscopy.

### 3.7 GC-MS analysis and proposed degradation mechanism

**Fig. 9:**
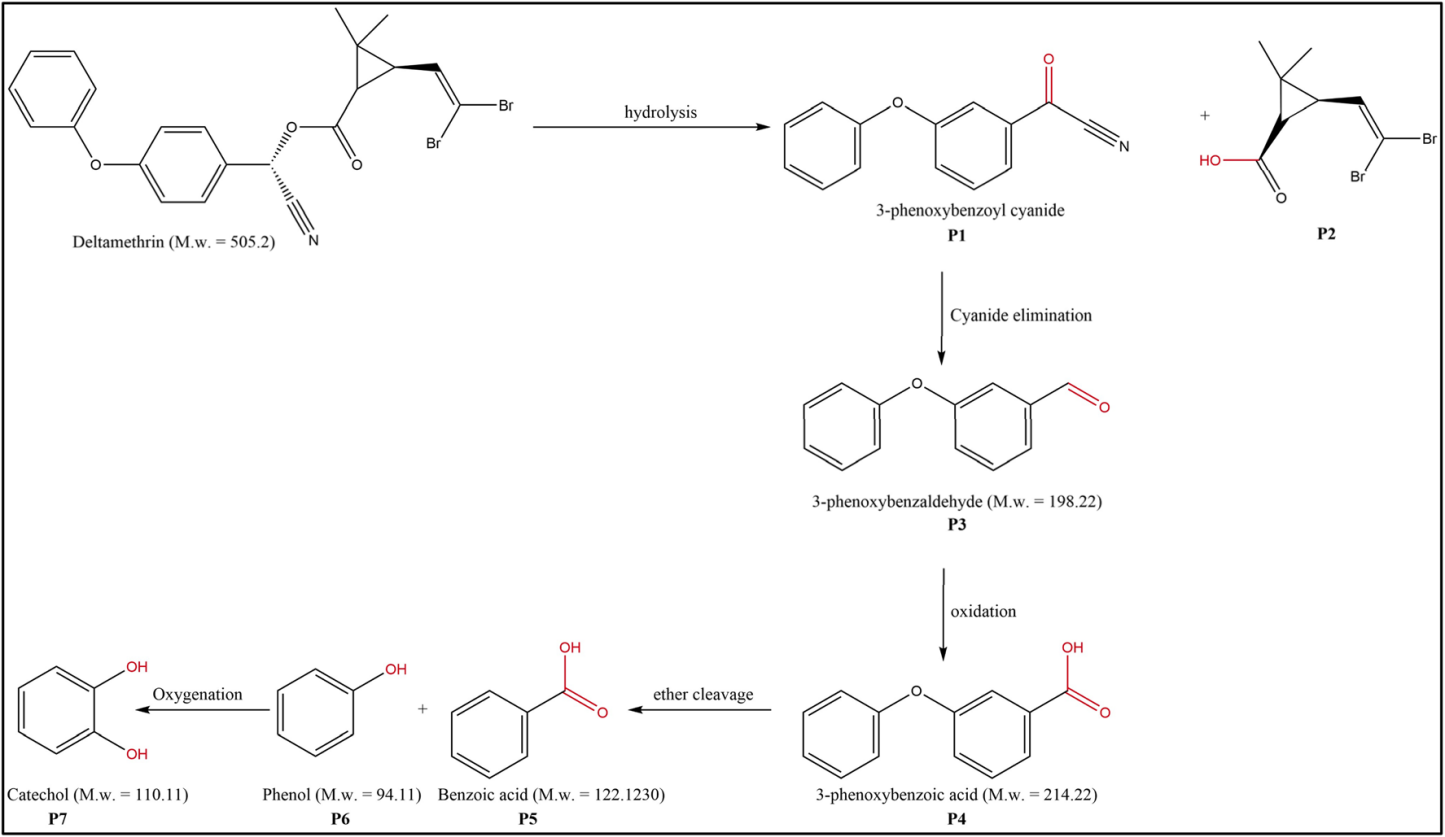
Possible degradation pathways of DELTA pesticide.

The intermediate molecules formed during the breakdown of DELTA by the ACP-DBD system were elucidated using GC-MS analysis, as shown in Fig. SE.3. The results confirm substantial molecular fragmentation and transformation of the parent pesticide. The process involves multiple sequential steps driven by interactions with RONS. The degraded molecules were identified by GC-MS, and subsequently, a degradation pathway was generated as shown in Fig. 9. Structurally, the DELTA molecule contains a cyclopropane ring, benzyl group, a cyano group and an ester linkage- of which the ester bond is particularly susceptible to attack.^47,48^ The initial degradation step involves hydrolysis of this ester linkage by ACP-DBD generated radicals, resulting in the formation of 3-phenoxybenzoyl cyanide (P1) and a cyclopropane-based acid fragment (P2). Subsequently, P1 undergoes cyanide elimination to yield phenoxy benzaldehyde (P3), a crucial intermediate which is further oxidized to 3-phenoxybenzoic acid (P4).^49,50^ The pathway then progresses through ether bond cleavage in the phenoxy moiety, forming benzoic acid (P5) and phenol (P6). Further oxidation of phenol leads to the generation of catechol (P7), indicating extensive oxidative transformation.

Similar degradation pathways have been reported in earlier studies by Aiello *et al.* 2020 and Tang *et al.* 2020.^47,51^ In the proposed degradation mechanism, the intermediate products show the disappearance of DELTA's characteristic peaks at retention times of about 27.19 and 27.43 minutes in the plasma-treated samples. New peaks corresponding to the identified degradation intermediates, such as 3-phenoxybenzaldehyde, 3-phenoxybenzoic acid, phenol, and catechol, have also emerged in the spectra after the cold plasma treatment. These results confirm the fragmentation and oxidation of the parent pesticide molecule.

FTIR spectroscopy also demonstrates significant structural alterations in DELTA with plasma treatment, which can be inferred from FTIR results. This degradation involves the oxidation hypothesis and oxidation due to the interaction of RONS. A noticeable increase in the O–H stretching band at 3436 cm^−1^ signifies the generation of hydroxyl-rich compounds, such as alcohols and carboxylic acids. The decline in the C–H stretching region around 2929.66 cm^−1^ indicates the degradation of aliphatic hydrocarbon chains. Additionally, the reduction in intensity at 1623 cm^−1^ (C=O and C=C stretching) and enhancement in the 1047.27 cm^−1^ region (C–O and C–O–C stretching) confirm the cleavage of aromatic rings and ether bonds, corroborating the GC-MS identification of phenolic and carboxylic by-products such as P5, P6 and P7.

Overall, the results provide a understanding of the ACP-DBD induced degradation pathway of DELTA. The parent pesticide is broken down into smaller, less hazardous, and environmentally safe compounds through hydrolysis, oxidation, cyanide removal, and ether bond scission. These results highlight the potential of ACP-DBD plasma as a sustainable and highly effective method for degrading pesticide residues in cocopeat-based agricultural systems.

### 3.8 Toxicity evaluation

The Toxicity evaluation software tool (T.E.S.T) was used to evaluate the biotoxicity (Daphnia magna LC_50_ and fathead minnow LC_50_) of DELTA and its degraded products (P0–P7). The LC_50_ endpoint for *Daphnia magna* represents the concentration of a test compound predicted to cause 50% mortality in this aquatic invertebrate after 48 hours of exposure. Similarly, the LC_50_, the endpoint for fathead minnows (*Pimephales promelas*), represented the estimated concentration responsible for 50% mortality in this freshwater vertebrate species over a 96-hour (4 days) period of exposure. As a standard model organism in aquatic toxicology, fathead minnow responses provide crucial data on the effects of chemical exposure on fish populations. Xu *et al.* (2023) and Mishra *et al.* (2025) and published similar toxicity prediction experiments using the T.E.S.T. Software, have been reported by further validating the utility of this tool in evaluating the ecological safety of degraded compounds.^27,52^

**Fig. 10:**
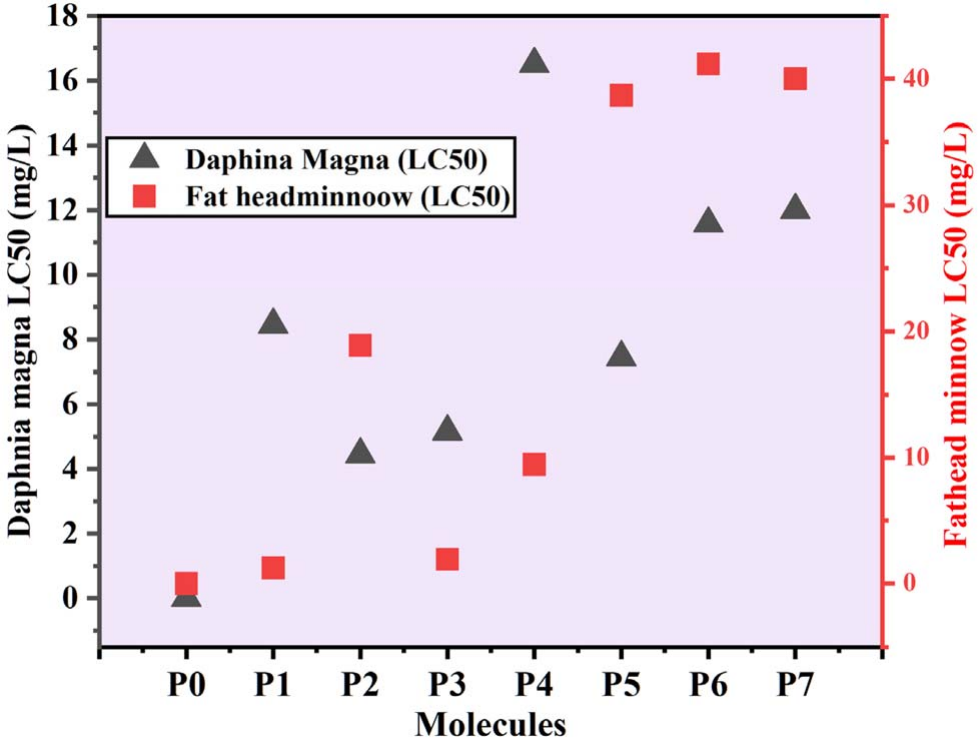
Predicted biological toxicity (LC_50_) of DELTA and its degraded metabolites using Toxicity Evaluation Software Tool (US EPA T.E.S.T.).

Together, the selected toxicity endpoints facilitate an integrated understanding of the potential environmental and health impacts posed by DELTA and its degradation products. The results shown in Fig. 10 illustrate that the biological toxicity of the seven degraded metabolites of DELTA (P1, P2, P3, P4, P5 P6 and P7) to *Daphnia magna* and fathead minnow is lower than that of DELTA itself. The biological toxicity of DELTA to daphnia and fathead is 0.00004 and 0.068 mg L^−1^, respectively. The toxicity predictions from Fig. 10 show that the biological toxicity of the seven degraded metabolites (P1, P2, P3, P4, P5, P6 and P7) is significantly lower than that of the parent compound, DELTA (P0), across all three test organisms. This indicates a positive trend toward detoxification following plasma treatment. *Daphnia magna* exhibited the highest sensitivity, with consistently lower LC_50_ values compared to the other organisms, identifying this species as a critical indicator for evaluating aquatic toxicity.

P0 (DELTA) had the highest acute toxicity among the evaluated compounds, with the lowest LC_50_ values in all test species, suggesting a high potential for both aquatic and terrestrial toxicity. On the other hand, P5 and P6 showed significantly lower toxicity, especially in fathead minnows, indicating a reduced ecological risk in aquatic environments. Compounds P3 and P4 showed the highest LC_50_ values in the daphnia. While the toxicity of the intermediate breakdown products of DELTA may vary, their overall toxicity throughout the process remains lower than that of DELTA itself. Similar results were previously reported by Xu *et al.* (2023).^52^ Overall, the results confirm that the environmental impact of DELTA can be significantly mitigated with ACP-DBD.

A comparison of different plasma reactors operated at different powers for the degradation of various pesticides is shown in Table 2. The results of this study demonstrate that the ACP-DBD system is highly effective in degrading DELTA in cocopeat soil with higher efficiency but with relatively less energy use. The degradation efficiency showed a strong dependence on the applied electrical parameters. Increasing discharge voltage and frequency enhanced micro discharges, thereby promoting the generation of RONS. These species, including ˙OH, O˙, O_3_, NO and NO_2_, played a central role in the oxidative decomposition of DELTA, consistent with the established roles of plasma-generated radicals in pesticide oxidation reported in earlier studies.^60^ The OES confirmed the presence of excited ˙OH, N_2_ and N_2_^+^ species, indicating the presence of an energetic environment for breaking chemical bonds, which is confirmed by the disappearance of characteristic DELTA peaks and formation of oxygenated intermediates in FTIR and GC-MS analysis. To further support the reliability of the plasma-induced degradation toxicity of the intermediated compound was generated.

**Table 2:** Comparison of various ACP-DBD and plasma-based soil remediation methods

DBD system	Pollutants in cocopeat soil	Treatment time (min)	Soil mass (g)	Degradation (%)	Energy (mg kJ^−1^)	Ref.
DBD, AC	Atrazine (100 mg kg^−1^), sandy cocopeat	60		84.8	0.30	53
Pulsed corona *ex situ*	Nitrophenol (silt loam)	60	10	82	0.14	54
Pulsed corona *ex situ*	Petroleum (5000 ppm) in silt loam	60	5	76.9	0.2	55
Pulsed DBD	Phenanthrene (200 ppm) in sandy soil	20	5	87.3	0.001	56
Pulsed corona *ex situ*	PFOA (30 ppm) in natural cocopeat soil	120	5	71	0.0039	57
ACP-DBD *ex situ*	Pyrene (105 ppm in loamy soil	30	5	79.7	0.0082	58
NSP-DBD in-cocopeat soil	Ciprofloxacin (200 ppm) in sandy soil	5	16.7	95.2	21.2	59
ACP-DBD	Deltamethrin in cocopeat soil (30 ppm)	15	10	84.18	0.33	This study

These simulated experiments showed a significant pesticide degradation in spiked cocopeat, while cocopeat enabled effective radical penetration due to its high porosity, natural soils are far more complex. To achieve similar pesticide decontamination, the soil profile plays a key role due to its wide mineralogy, organic content, pH variations, and microbial profile.^61^ Therefore, future studies should focus on plasma applications in diverse soil matrices to evaluate soil-plasma interactions for a deeper understanding of radical transport and reaction pathways in different soil compositions, textures and contaminant breakdown. Although this study predicted reduced toxicity of DELTA degraded intermediates, their long-term persistence, mobility and interactions with soil components remain unexplored. The long-term effectiveness of plasma-treated soil and ecotoxicity assays is essential to ensure plasma treatments effect on soil ecological balance.^62^ Similarly, the repeated plasma exposure to soil nutrients and microbial populations required further investigation. Additionally, the transition from lab scale to pilot scale and further to field scale application of plasma presents several challenges. This requires uniform plasma discharge over a large surface area under various environmental conditions, while maintaining energy efficiency for large-scale operations. It is recommended to develop a hybrid plasma-catalyst design with real-time diagnostics, such as an *in situ* RONS sensor, for real-world field applications. These investigations will provide insights into the concept of process scalability and stability in practical applications. These improvements will result in the real-world use of plasma-based soil remediations in the field, providing chemical- free and sustainable farming methods.

## 4. Conclusion

This study shows that treating pesticide-contaminated soils with ACP-DBD plasma is a very promising and sustainable method. The degradation efficiency of DELTA was significantly influenced under optimized conditions, reaching up to 71.22% to 84.5% after 15 minutes of plasma treatment. The system demonstrated performance across diverse pollutant concentrations, moisture contents and soil matrices, highlighting its versatility. The breakdown and removal of DELTA were also clearly influenced by the pH of the soil. Alkaline conditions and high moisture were beneficial to the removal of DELTA at optimized conditions. Several degradation intermediates were identified by FTIR and GC-MS characterization, indicating a reaction pathway primarily influenced by ^1^O_2_ and hydroxyl radicals. These findings are consistent with well-established plasma chemistry and align with the understanding that pesticide breakdown is driven by strong oxidative transformation routes. Moreover, the technique's superior energy efficiency and adaptability to heterogeneous soil conditions affirm its practical applicability. Collectively, ACP-DBD emerges as an eco-friendly, cost-effective alternative to conventional soil remediation approaches. Future research should focus on reactor scale-up and continuous operation modalities to facilitate large-scale implementation in agricultural and environmental remediation.

## Author contributions

Sushma Jangra: conceptualization (lead); investigation (equal); methodology (lead); data curation (equal); writing – original draft (lead); writing – review editing(supporting). Abhijit Mishra: formal analysis (equal); investigation (supporting); writing – original draft (supporting); data curation(equal); Ritesh Mishra: formal analysis (supporting); writing – review & editing (supporting); data curation (supporting), methodology (supporting); investigation (supporting); Shikha Pandey: formal analysis (supporting); investigation (supporting); validation (supporting). Ram Prakash: writing – review & editing (lead), conceptualization (equal); supervision (lead); resources (lead); funding acquisition (lead).

## Conflicts of interest

The authors declare that they have no known competing financial interests or personal relationships that could have appeared to influence the work reported in this paper.

## Abbreviations

ACP: Atmospheric pressure cold
DBD: Dielectric barrier discharge
FESEM: Field emission scanning electron microscopy
OES: Optical emission spectroscopy
RONS: Reactive oxygen-nitrogen species
DELTA: Deltamethrin

## Supplementary Material

RA-016-D5RA08882H-s001

## Data Availability

Data will be made available on request. Supplementary information (SI) is available. See DOI: https://doi.org/10.1039/d5ra08882h.
