# Supplementary material for: Effective degradation of DELTA pesticide in soil by an atmospheric-pressure cold plasma-DBD system
Source: RSC Adv. 2026 Jan 12;16(3):2585–96. doi: 10.1039/d5ra08882h (PMC12794205; doi:10.1039/d5ra08882h)
Supplement: RA-016-D5RA08882H-s001 [file RA-016-D5RA08882H-s001.pdf]

Supplementary Information (SI) for RSC Advances.
This journal is © The Royal Society of Chemistry 2025

Effective Degradation of DELTA Pesticide in Soil by Atmospheric-pressure Cold Plasma-DBD System

Sushma Jangra[1], Abhijit Mishra[1], Ritesh Mishra[2], Shikha Pandey[1] and Ram Prakash[1,2]

Department of Physics, Indian Institute of Technology Jodhpur, Rajasthan, India, 342030

[2]Interdisciplinary Research Division- Smart Healthcare, Indian Institute of Technology Jodhpur, Rajasthan, India, 342030

E-mail address: ramprakash@iitj.ac.in

SE.1 Sampling Procedures.

For neutral and alkaline cocopeat soil, all the soil samples were mixed with 50 mL NaOH solution (pH = 11.0) in airtight sealed conical flask and then shaken on a shaker at 300 rpm for 12 h to extract DELTA from the soil samples, then the mixture was centrifuged at 3000 rpm for 15 min. After centrifugation the extract was passed through a 0.45 µm filter, 1 mL filtrate was used for DELTA analysis, A 1 mL aliquot of the filtrate was collected for DELTA analysis. For acidic soils (HCl-treated), the extraction was performed using 50 mL of methanol instead of NaOH under identical conditions. The samples were shaken at 300 rpm for 12 hours in sealed conical flasks, followed by centrifugation at 3000 rpm for 15 minutes. The resulting extracts were similarly filtered through a 0.45 µm membrane filter, and 1 mL of the filtrate was used for further DELTA analysis.

SE.2 High Performance Liquid Chromatography (HPLC) analysis

(a)

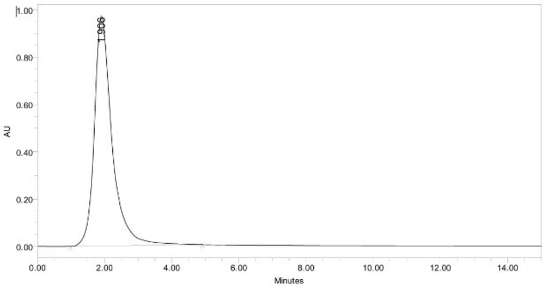

	RT	Area	% Area	Height
1	1.906	35765937	100.00	972635

Fig. SE.2 (a) Untreated sample of soil

(b)

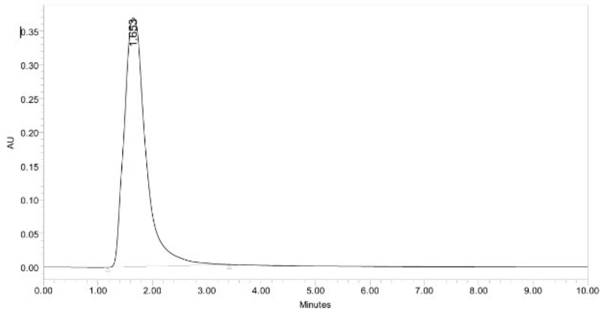

	RT	Area	% Area	Height
1	1.653	10197978	100.00	369028

Fig. SE.2 (b) 5 min treated sample soil

(c)	(d)
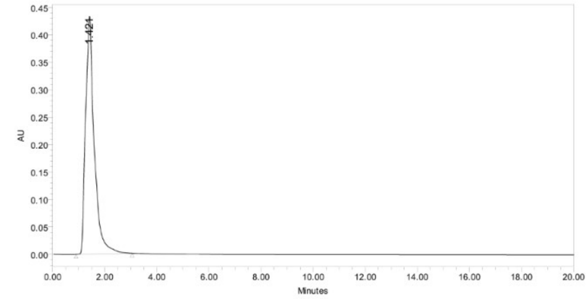	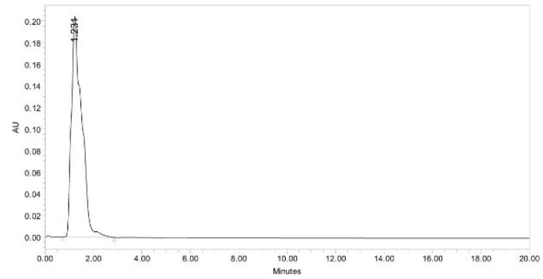

	RT	Area	% Area	Height
1	1.421	9978073	100.00	433593

	RT	Area	% Area	Height
1	1.231	6027958	100.00	204436

Fig. SE.2 (c)10 min plasma treated sample

Fig. SE.2 (d) 15 min plasma treated sample

SE.3 Gas Chromatography Mass Spectrometry (GC-MS) analysis

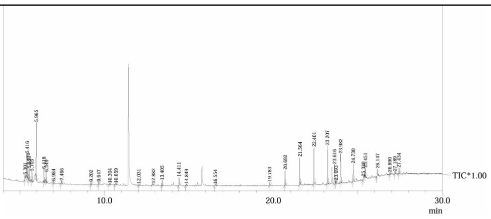

Peak Report TIC

Peak#	R.Time	Area	Area%	Name
1	5.301	49597	0.80	2,6-ETHENOOXIRENO[4',5']BENZO[1',2':3,4]CYCLOBU
2	5.416	316221	5.09	Benzene, 1-ethyl-3-methyl-
3	5.470	165415	2.67	Benzene, 1-ethyl-4-methyl-
4	5.553	165772	2.67	BENZENE, 1,3,5-TRIMETHYL-
5	5.705	132955	2.14	Benzene, 1-ethyl-4-methyl-
6	5.965	748649	12.06	Benzene, 1,2,3-trimethyl-
7	6.418	173575	2.80	BENZENE, 1,3,5-TRIMETHYL-
8	6.549	143708	2.32	CYCLOHEXENE, 1-METHYL-4-(1-METHYLETHENYL
9	6.984	38978	0.63	Benzene, tert-butyl-
10	7.466	86945	1.40	DELTA-2-CARENE
11	9.202	46989	0.76	PENTADECANE
12	9.647	40091	0.65	2Z,6E-FARNESOL
13	10.304	21426	0.35	DOCOSANE
14	10.659	23756	0.38	Sulfurous acid, 2-ethylhexyl hexyl ester
15	12.031	16147	0.26	DOCOSANE
16	12.882	39960	0.64	3-Ethoxy-1,1,1,7,7,7-hexamethyl-3,5,5-tris(trimethylsiloxy)
17	13.405	77618	1.25	Phenol, 3,5-bis(1,1-dimethylethyl)-
18	14.411	175592	2.83	1,2-BENZENEDICARBOXYLIC ACID, DIETHYL ESTE
19	14.849	31995	0.52	3,4-DIHYDROXYMANDELIC ACID-TETRATMS
20	16.554	35972	0.58	1,3-Dioxolane, 2-(phenylmethyl)-
21	19.783	63523	1.02	TETRACOSANE
22	20.692	245883	3.96	Eicosane
23	21.564	438804	7.07	Heneicosane
24	22.401	598370	9.64	Tetratetracontane
25	23.207	565025	9.10	Tetratetracontane
26	23.616	350521	5.65	2,5-Dimethyl-4-benzyl-pyridine
27	23.693	33210	0.54	Eicosane, 1-iodo-
28	23.982	459680	7.41	Hexatriacontane
29	24.730	367357	5.92	Nonacosane
30	25.330	71087	1.15	Palmitoleamide
31	25.451	117297	1.89	Eicosane
32	26.147	156887	2.53	Dotriacontane, 1-iodo-
33	26.890	37281	0.60	Carbonic acid, eicosyl vinyl ester
34	27.189	48805	0.79	Deltamethrin
35	27.434	121662	1.96	Deltamethrin
		6206753	100.00	

Fig. SE.3 (a) Untreated soil

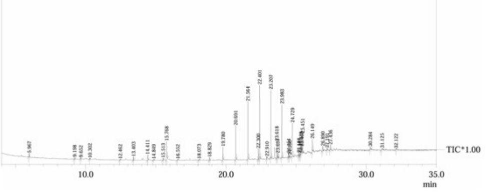

Peak Report TIC

Peak#	R.Time	Area	Area%	Name
1	5.967	60404	0.62	BENZENE, 1,2,3-TRIMETHYL-
2	9.198	40967	0.42	OCTADECANE, 2-METHYL-
3	9.652	24378	0.25	Cyclopentane, nitro-
4	10.302	13231	0.14	DOCOSANE
5	12.462	24614	0.25	1-Oxaspiro[2.5]octane, 4,4-dimethyl-8-methylene-
6	13.403	63719	0.66	Phenol, 3,5-bis(1,1-dimethylethyl)-
7	14.411	146470	1.51	1,2-BENZENEDICARBOXYLIC ACID, DIETHYL ESTE
8	14.849	28205	0.29	BENZOIC ACID, 2,5-BIS(TRIMETHYLSILOXY)-, TRIM
9	15.513	41817	0.43	2-NORPINENE, 2,6-DIMETHYL-6-(4-METHYL-3-PENT
10	15.768	433833	4.49	Benzaldehyde, 3-phenoxy-
11	16.552	27540	0.28	Benzoic acid, [4-(thietan-3-yloxy)benzylidene]hydrazide
12	18.073	32940	0.34	3-Pentanol, 3-methyl-
13	18.829	58103	0.60	Sulfurous acid, 2-ethylhexyl hexyl ester
14	19.780	231816	2.40	Heneicosane
15	20.691	633772	6.55	Heneicosane
16	21.564	1066563	11.03	Heneicosane
17	22.300	207530	2.15	HEXANEDIOIC ACID, BIS(2-ETHYLHEXYL) ESTER
18	22.401	1399678	14.47	Heneicosane
19	22.910	24531	0.25	TETRACOSANE
20	23.207	1285174	13.29	Eicosane
21	23.618	376044	3.89	2,5-Dimethyl-4-benzyl-pyridine
22	23.697	85479	0.88	Octacosane, 2-methyl-
23	23.983	1025186	10.60	Tetratetracontane
24	24.454	70794	0.73	Octacosane, 2-methyl-
25	24.530	39192	0.41	Eicosane, 1-iodo-
26	24.729	756498	7.82	Hexatriacontane
27	25.184	47576	0.49	Dotriacontane, 1-iodo-
28	25.262	47241	0.49	Eicosane, 1-iodo-
29	25.328	213365	2.21	13-Docosenamide, (Z)-
30	25.402	64563	0.67	3,5-Dimethyl-1-diphenylmethylsilyloxybenzene
31	25.451	304644	3.15	Hexatriacontane
32	26.149	293525	3.04	Triacontane, 1-iodo-
33	26.890	111865	1.16	2-Methylhexacosane
34	27.191	43127	0.45	Deltamethrin
35	27.436	146216	1.51	Deltamethrin
36	30.284	87430	0.90	.beta.-Sitosterol
37	31.125	93464	0.97	Phenol, 2,4-bis(1,1-dimethylethyl)-, phosphite (3:1)
38	32.122	18768	0.19	Tricyclo[3.1.0.0(2,4)]hexane, 3,3,6,6-tetraethyl-,trans--
		9670262	100.00	

Fig. SE.3 (b) 15 minutes plasma treated soil
